# Digital single-operator cholangioscope-assisted endoscopic retrograde appendicitis therapy in the management of Crohnʼs disease with acute appendicitis

**DOI:** 10.1055/a-2410-3702

**Published:** 2024-09-19

**Authors:** Dezheng Lin, Mingli Su, Yuping Su, Zehui Guo, Jiaxin Deng, Yongcheng Chen, Xuefeng Guo

**Affiliations:** 1 373651 Department of Endoscopic Surgery, Department of General Surgery, The Sixth Affiliated Hospital, Sun Yat-sen University, Guangzhou, Guangdong, China; 2 373651 Guangdong Provincial Key Laboratory of Colorectal and Pelvic Floor Diseases, The Sixth Affiliated Hospital, Sun Yat-sen University, Guangzhou, Guangdong, China; 3 373651 Biomedical Innovation Center, The Sixth Affiliated Hospital, Sun Yat-sen University, Guangzhou, Guangdong, China

**Fig. 1:**
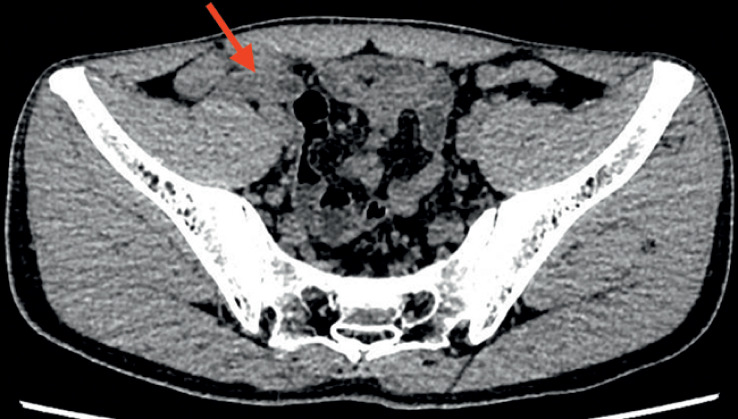
Computed tomography scan image showing acute suppurative appendicitis, with an enlarged appendix (arrow).

A 30-year-old man was admitted with right lower abdominal pain for 2 days. A computed tomography scan showed acute suppurative appendicitis with an enlarged appendix ( Fig. 1 ). At the time, the patient was experiencing an active phase of Crohnʼs disease and was undergoing treatment with ustekinumab, making appendectomy inadvisable. He therefore underwent direct vision endoscopic retrograde appendicitis therapy (ERAT).

**Fig. 2:**
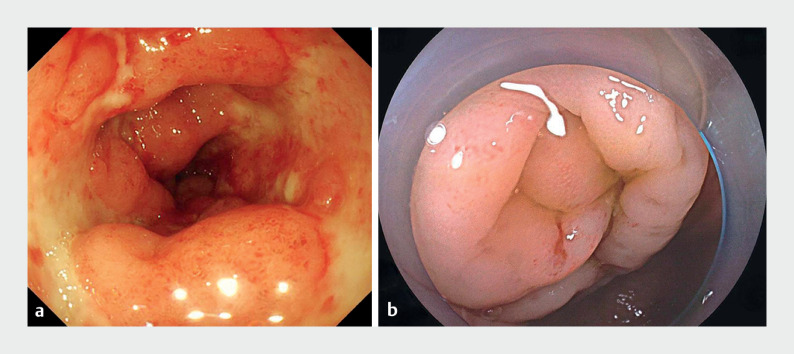
Colonoscopic images showing: **a** evidence of active Crohnʼs disease; **b** the appendiceal orifice.

**Fig. 3:**
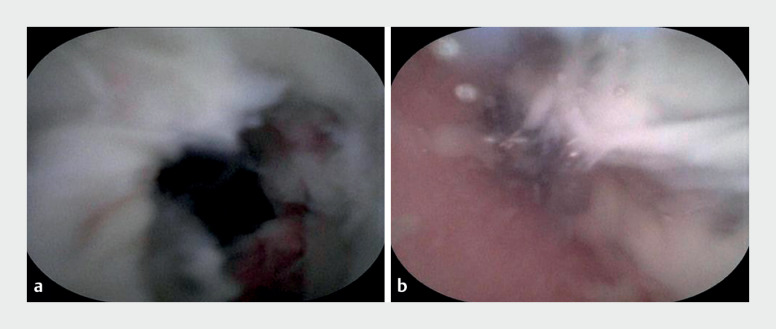
Cholangioscopic image showing: **a** the mucosa within the appendiceal cavity; **b** white purulent discharge in the appendiceal cavity.

**Fig. 4:**
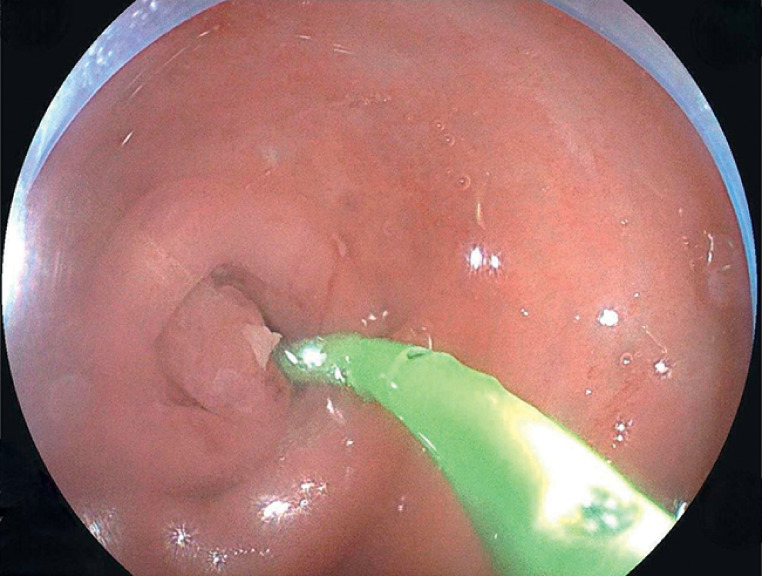
Endoscopic image showing a single-pigtail pancreatic stent inserted into the appendix.

Colonoscopy revealed a cobblestone-like appearance of the colonic mucosa, with scattered irregular superficial ulcers covered by a white exudate ( Fig. 2 **a** ), confirming the diagnosis of active Crohnʼs disease. Once the cecum had been reached, the mucosa of the appendiceal orifice appeared edematous ( Fig. 2 **b** ). A digital single-operator cholangioscope (eyeMax; Micro-Tech, Nanjing, China), inserted into the appendiceal cavity, was used to perform appendicoscopy ( Video 1 ). Direct visualization provided a clear view of congestion and edema within the appendiceal cavity ( Fig. 3 **a** ). It also revealed expansion of the lumen and a significant amount of white purulent discharge ( Fig. 3 **b** ). The appendix was irrigated with metronidazole sodium solution, and a single-pigtail pancreatic stent was placed with guidewire assistance ( Fig. 4 ). Post-ERAT, the patient rapidly experienced pain relief and was discharged without complications.

A digital single-operator cholangioscope is used to perform appendicoscopy and endoscopic retrograde appendicitis therapy (ERAT).Video 1

For patients with Crohnʼs disease with severe complications, such as intestinal perforation, persistent or recurrent intestinal obstruction, abdominal abscess not amenable to percutaneous drainage, refractory gastrointestinal bleeding, dysplasia, or cancer, surgical intervention is required. Surgical interventions can potentially result in significant complications, an elevated risk of recurrence, and a diminished quality of life. Direct vision ERAT is an effective treatment for appendicitis. To the best of our knowledge, this is the first report of the use of a digital single-operator cholangioscope to treat Crohnʼs disease with acute appendicitis. In such a situation, this method avoids the need for appendectomy and the potential complications associated with the surgery.

Endoscopy_UCTN_Code_TTT_1AQ_2AF

